# Modelling the impact of birth control policies on China’s population and age: effects of delayed births and minimum birth age constraints

**DOI:** 10.1098/rsos.211619

**Published:** 2022-06-08

**Authors:** Yue Wang, Renaud Dessalles, Tom Chou

**Affiliations:** ^1^ Department of Computational Medicine, UCLA, Los Angeles, CA 90095-1766, USA; ^2^ Greenshield, 46 rue Saint Antoine, 75004 Paris, France; ^3^ Department of Mathematics, UCLA, Los Angeles, CA 90095-1555, USA

**Keywords:** population biology, demographics, McKendrick equation, population control, one-child policy

## Abstract

We consider age-structured models with an imposed refractory period between births. These models can be used to formulate alternative population control strategies to China’s one-child policy. By allowing any number of births, but with an imposed delay between births, we show how the total population can be decreased and how a relatively older age distribution can be generated. This delay represents a more ‘continuous’ form of population management for which the strict one-child policy is a limiting case. Such a policy approach could be more easily accepted by society. Our analyses provide an initial framework for studying demographics and how social constraints influence population structure.

## 1. Introduction

Models of age-structured population dynamics are often based on the classic McKendrick equation [1,2] (sometimes called the von Foerster equation [3]). These equations describe the dynamics of the mean population as a function of time *t* and expressed as a density in age *a*. The solutions to the McKendrick equations can be partially solved using the method of characteristics and numerical approximations [4,5] across many contexts. Moreover, stochastic extensions to incorporate the random times of birth and death (demographic stochasticity) have been formulated using branching processes [6] and kinetic and operator theory [7–10].

**Figure 1.**
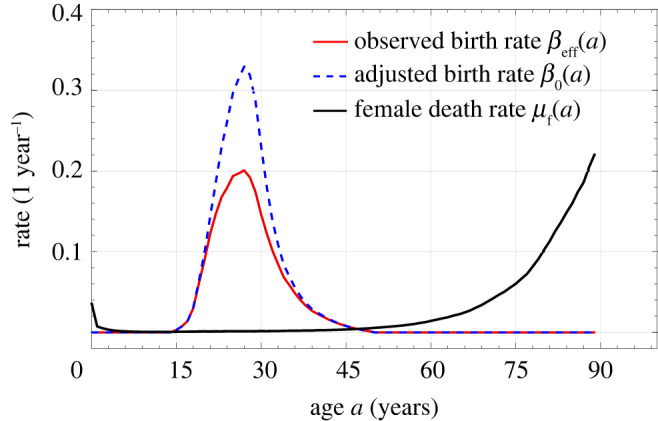
China’s 1981 birth rate and female death rate *μ*_f_(*a*), calculated from 1982 national census data [20]. The red curve represents the observed birth rate *β*_eff_(*a*) for all women of a given age. The dashed blue curve represents the birth rate *β*_0_(*a*) for females who have not had any children. The area under the observed birth rate $\int_{0}^{\infty }\beta_{\mathrm{eff}}(a)da$ represents the mean number of children born during an individual’s lifetime.

Age-structured equations have been used to predict the evolution of human and animal populations [11–13]. Using such models and ideas from control theory to frame population control strategies was in vogue in the 1970s [14–17]. A profound example was its use in 1979 by Jian Song [18,19], a Chinese engineer who numerically solved the one-component McKendrick equation using birth rates associated with China in the late 1970s (figure 1). By projecting future populations associated with different birth rates (expressed by the mean number of children per woman), he found that in order to keep the population manageable (approx. 700 million−1 billion) within 100 years, this control parameter would have to be decreased to the point where each woman is allowed only one child [18,19,21]. This research provided the technical basis of the one-child policy in China [22,23].

In the 1970s, China had encouraged (but not enforced) people to marry later, wait longer before childbearing, and have fewer children (‘later-longer-fewer’ policy) [11]. Despite concerns from social scientists and demographers who proposed such ‘softer’ controls, the one-child policy was implemented in 1980, based on the implications of Jian Song’s numerical solutions to the McKendrick equation. Rather than imposing a maximal number of children, a minimum delay between two consecutive births [23] or a minimum birth age could have been imposed. Such a policy would arguably have been more easily enforced and would have led to fewer unintended consequences such as a skewed sex ratio and an elder-heavy age distribution. Here, we retrospectively model such alternatives and make predictions as policies change.

Specifically, we extend the McKendrick age-structured model to incorporate a delay between successive births by each female. In order to do so, we must explicitly delineate individuals who have not given birth from those who have given birth at least once. Imposed delays between successive births can then be formally described by adjusting the birth rate function of the individuals who have given birth at least once. We solve our model equations using parameters appropriate to 1981 China and compare predictions of the graded policies with those of a strict one-child policy. We explore how the total population and age distribution are affected by different values of imposed refractory periods and minimum birth age.

## 2. Mathematical model

When applying age-structured partial differential equation (PDE) models to two-sex populations, a simple assumption is to consider only the density of females at time *t* with age *a*. The predicted number of females with age between *a* and *a* + d*a* is thus *f*(*a*, *t*)d*a*. Indeed, unless the female population is much larger than the male population (e.g. after a war), the female population can be considered as the ‘limiting quantity’ that determines the number of births. In other words, the frequency of births in the total population is relatively insensitive to the male population. The McKendrick equations describing the female population density *f*(*a*, *t*) are formulated as

2.1*a* $$\frac{\partial }{\partial t}f(t,a)+\frac{\partial }{\partial a}f(t,a)=-\mu_{f}(a)f(t,a),$$

2.1*b* $$f(t,0)=\eta \int_{0}^{\infty }\;\beta_{\mathrm{eff}}(a)f(t,a)da$$

2.1*c* $$\;\mathrm{and}\;f(0,a)=I_{f}(a),$$

where *μ*_f_(*a*) represents the death rate of females of age *a*, *β*_eff_(*a*) is the observed birth rate of women of age *a*, *η* is the fraction of births that produce girls, and *I*_f_(*a*) is the age distribution of the initial population at *t* = 0.

Equation (2.1*a*) describes the time-evolution of the population, equation (2.1*b*) denotes the boundary condition at age *a* = 0 describing the number of girls born at time *t*, and equation (2.1*c*) specifies the initial condition. This model neglects the explicit mating-age male population which is valid when *η* is maintained below 0.5, giving rise to more males than females. For humans, *η* ≈ 0.48 − 0.49 naturally (but this is compensated by a slightly higher mortality in males across all ages). With sex-selective abortion, *η* can be even smaller [24]. If one were also interested in the male population *m*(*t*, *a*), it would obey the same equations except with a male version of the death rate *μ*_m_(*a*), an initial condition *I*_m_(*a*), and a boundary condition for male newborns: $m(t,0)=(1-\eta )\int_{0}^{\infty }\beta_{\mathrm{eff}}(a)f(t,a)da$.

### 2.1. Delayed birth model

Now, in order to introduce a delay between consecutive births, we need to further partition the female population into those who have never had a child and those who have already had a child (and who may need to wait a certain time before having another one). The population densities for each of these classes of females are defined as:

*f*_0_(*t*, *a*): the population density of childless females. The quantity *f*_0_(*t*, *a*)d*a* is the number of females with age between *a* and *a* + d*a* and who have never had a child up to the current time *t*.
*f*(*t*, *a*, *τ*): the population of females who have had at least one child. The quantity *f*(*t*, *a*, *τ*)d*a*d*τ* is the number of females at time *t* whose age is between *a* and *a* + d*a* and whose youngest child’s age is between *τ* and *τ* + d*τ*.

We will assume that these two populations have the same age-dependent death rate *μ*_f_(*a*) but give birth at different rates *β*_0_(*a*) and *β*(*a*, *τ*), respectively. We also define the total female population density as

2.2 $$f_{\mathrm{tot}}(t,a)=f_{0}(t,a)+\int_{0}^{a}\;f(t,a,\tau )\;d\tau ,$$

and the total number of females at time *t* as

2.3 $$n(t)=\int_{0}^{\infty }\;f_{\mathrm{tot}}(t,a)da=\int_{0}^{\infty }\;f_{0}(t,a)da+\int_{0}^{\infty }\;da\int_{0}^{a}\;d\tau f(t,a,\tau ).$$

The age-structured McKendrick equations for *f*_0_ and *f* are:

2.4*a* $$\frac{\partial }{\partial t}f_{0}(t,a)+\frac{\partial }{\partial a}f_{0}(t,a)=-(\mu_{f}(a)+\beta_{0}(a))f_{0}(t,a),$$

2.4*b* $$\frac{\partial }{\partial t}f(t,a,\tau )+\frac{\partial }{\partial a}f(t,a,\tau )+\frac{\partial }{\partial \tau }f(t,a,\tau )=-(\mu_{f}(a)+\beta (a,\tau ))f(t,a,\tau ),$$

2.4*c* $$f_{0}(t,0)=\eta \left(\int_{0}^{\infty }\;\beta_{0}(a)f_{0}(t,a)da+\int_{0}^{\infty }\;da\int_{0}^{a}\;d\tau \;\beta (a,\tau )f(t,a,\tau )\right),$$

2.4*d* $$f(t,a,0)=\beta_{0}(a)f_{0}(t,a)+\int_{0}^{a}\;\beta (a,\tau )f(t,a,\tau )\;d\tau$$

2.4*e* $$f_{0}(0,a)=I_{0}(a)\;\text{and}\;f(0,a,\tau )=I(a,\tau ).$$

Equation (2.4*a*) describes the evolution of *f*_0_ as in the classical McKendrick equation (cf. equation (2.1*a*)) with birth rate *β*_0_(*a*). For *f*(*t*, *a*, *τ*), we must introduce the new variable *τ* to mark the time since the last birth. This brings in another convection term in equation (2.4*b*) since *τ* increases alongside time *t* and age *a*. The birth rate *β* for this population can depend on both the age *a* and the time *τ* since the last birth. Equation (2.4*c*) gives the number of girls *f*_0_(*t*, 0) born at time *t*, while equation (2.4d) describes *f*(*t*, *a*, 0), the density of females at age *a* at time *t* who just gave birth. These individuals can arise from the *f*_0_ population (females who have never had a child) or from the *f* population itself (females who have already had at least one child). Thus, the boundary conditions (2.4*c*) and (2.4*d*) couple the two populations *f*_0_ and *f*. Finally, equations (2.4*e*) simply describe the initial conditions for *f*_0_ and *f*.

In appendix A, we explicitly show that the total female population density *f*_tot_(*t*, *a*) (equation (2.2)) satisfies the standard age-structured McKendrick equation

2.5 $$\frac{\partial }{\partial t}f_{\mathrm{tot}}(t,a)+\frac{\partial }{\partial a}f_{\mathrm{tot}}(t,a)=-\mu_{f}(a)f_{\mathrm{tot}}(t,a).$$

Within a model that explicitly considers the time *τ* since the last childbirth, we can easily describe an imposed hypothetical policy that applies a refractory period *δ* between births. After having a child and before this refractory period (0 ≤ *τ* ≤ *δ*) expires, the birth rate *β*(*a*, *τ*) can be set to 0. As a preliminary description, we will consider a policy-modified (truncated) birth rate function

2.6 $$\beta (a,\tau )=\beta_{0}(a)1(\tau ,\delta ),$$

where the indicator function 1(τ,δ)=1 for *τ* > *δ* and 1(τ,δ)=0 for *τ* ≤ *δ*. This form assumes that once the imposed refractory period has passed, the birth rate immediately rises back to a value associated with the person's current age.

### 2.2. Asymptotic behaviour

We first analyse the asymptotic behaviour of our model. An important feature of renewal transport equations such as the McKendrick model is that as *t* → ∞, the total population *n*(*t*) will grow exponentially (in the absence of nonlinear regulation terms [25]), while the normalized, age-dependent population density converges to a time-independent stationary distribution (see Perthame [5], ch. 3 and Arino [26]). This property is independent of the initial condition. We will assume that this steady-state asymptotic property arises in our two-component, three-variable model; i.e. the normalized densities *f*_0_(*t*, *a*)/*n*(*t*) and *f*(*t*, *a*, *τ*)/*n*(*t*) converge to stationary distributions. We denote the stationary limits as

2.7 $$\lim_{t\to \infty }\frac{\;f_{0}(t,a)}{n(t)}\equiv h_{0}(a)\;\text{and}\;\lim_{t\to \infty }\frac{\;f(t,a,\tau )}{n(t)}\equiv h(a,\tau ).$$

We also define the distribution associated with the total female population as

2.8 $$\lim_{t\to \infty }\frac{\;f_{\mathrm{tot}}(t,a)}{n(t)}\equiv h_{\mathrm{tot}}(a)=h_{0}(a)+\int_{0}^{a}\;h(a,\tau )\;d\tau ,$$

where $\int_{0}^{\infty }h_{\mathrm{tot}}(a)da=1$. If we assume that *f*_0_(0, *a*)/*n*(0) = *h*_0_(*a*) and *f*(0, *a*, *τ*)/*n*(0) = *h*(*a*, *τ*) for any *a*, *τ* at some initial time *t* = 0, then *f*_0_(*t*, *a*)/*n*(*t*) = *h*_0_(*a*) and *f*(*t*, *a*, *τ*)/*n*(*t*) = *h*(*a*, *τ*) hold for any *t* ≥ 0.

From equation (2.4*a*), we have

2.9 $$\begin{array}{rl} \frac{1}{f_{0}(t,a)}\frac{\partial f_{0}(t,a)}{\partial t} & =-\frac{1}{f_{0}(t,a)}\frac{\partial f_{0}(t,a)}{\partial a}-\mu_{f}(a)\\ & =-\frac{n(t)}{f_{0}(t,a)}\frac{1}{n(t)}\frac{\partial f_{0}(t,a)}{\partial a}-\mu_{f}(a)\\ & =\frac{1}{h_{0}(a)}\frac{dh_{0}(a)}{da}-\mu_{f}(a). \end{array}$$

Thus, [∂*f*_0_(*t*, *a*)/∂*t*]/*f*_0_(*t*, *a*) is independent of *t*. Moreover, for any *a*, *a*′, *τ*

2.10 $$\frac{\;f_{0}(t,a)}{f(t,a^{\prime },\tau )}=\frac{h_{0}(a)}{n(t)}\frac{n(t)}{h(a^{\prime },\tau )}=\frac{h_{0}(a)}{h(a^{\prime },\tau )},$$

is also independent of *t* so that

2.11 $$\frac{\partial }{\partial t}\left[\frac{\;f_{0}(t,a)}{f(t,a^{\prime },\tau )}\right]=\frac{1}{f{\left(t,a^{\prime },\tau \right)}^{2}}\left[\;f(t,a^{\prime },\tau )\frac{\partial }{\partial t}f_{0}(t,a)-f_{0}(t,a)\frac{\partial }{\partial t}f(t,a^{\prime },\tau )\right]=0.$$

Thus, for any *a*, *a*′, *τ*,

2.12 $$\frac{1}{f_{0}(t,a)}\frac{\partial f_{0}(t,a)}{\partial t}=\frac{1}{f(t,a^{\prime },\tau )}\frac{\partial f(t,a^{\prime },\tau )}{\partial t}=\frac{1}{f_{0}(t,a^{\prime })}\frac{\partial f_{0}(t,a^{\prime })}{\partial t}.$$

Equations (2.9) and (2.12) show that [∂*f*_0_(*t*, *a*)/∂*t*]/*f*_0_(*t*, *a*) is independent of both *t* and *a*, allowing us to define a constant that describes the stationary growth rate

2.13 $$\lambda =\frac{1}{f_{0}(t,a)}\frac{\partial f_{0}(t,a)}{\partial t}=\frac{1}{f(t,a,\tau )}\frac{\partial f(t,a,\tau )}{\partial t}=\frac{1}{f_{\mathrm{tot}}(t,a)}\frac{\partial f_{\mathrm{tot}}(t,a)}{\partial t}.$$

Thus, we can express solutions for the densities *f*_0_(*t*, *a*) and *f*(*t*, *a*, *τ*) in the form

2.14 $$f_{0}(t,a)=Ch_{0}(a)\;e^{\lambda t}\;\text{and}\;f(t,a,\tau )=Ch(a,\tau )\;e^{\lambda t},$$

where *C* is a constant. After using these expressions in equations (2.4), we find the equations for the stationary distributions

2.15*a* $$\frac{d}{da}h_{0}(a)=-(\mu_{f}(a)+\beta_{0}(a)+\lambda )h_{0}(a),$$

2.15*b* $$\frac{\partial }{\partial a}h(a,\tau )+\frac{\partial }{\partial \tau }h(a,\tau )=-(\mu_{f}(a)+\beta (a,\tau )+\lambda )h(a,\tau ),$$

2.15*c* $$h_{0}(0)=\eta \left(\int_{0}^{\infty }\beta_{0}(a)h_{0}(a)\;da+\int_{0}^{\infty }\;da\int_{0}^{a}\;d\tau \;\beta (a,\tau )h(a,\tau )\right)$$

2.15*d* $$\;\mathrm{and}\;h(a,0)=\beta_{0}(a)h_{0}(a)+\int_{0}^{a}\;\beta (a,\tau )h(a,\tau )\;d\tau .$$

Next, using equation (2.5), we find

2.16 $$\frac{d}{da}h_{\mathrm{tot}}(a)=-(\mu_{f}(a)+\lambda )h_{\mathrm{tot}}(a),$$

which is solved by

2.17 $$h_{\mathrm{tot}}(a)=h_{\mathrm{tot}}(0)\exp\left[-a\lambda -\int_{0}^{a}\;\mu_{f}(a^{\prime })\;da^{\prime }\right].$$

We can then define the effective whole-population birth rate function

2.18 $$\beta_{\mathrm{eff}}(a)=\frac{\beta_{0}(a)h_{0}(a)+\int_{0}^{a}\;\beta (a,\tau )h(a,\tau )\;d\tau }{h_{\mathrm{tot}}(a)},$$

which describes the overall birth rate weighted over the entire stationary population. This population-averaged birth rate *β*_eff_(*a*) corresponds to that used in the basic lumped model (equation (2.1*b*)) and is the quantity that can be directly extracted from birth data that provide women’s ages at time of birth, but that may not distinguish whether females are first-time mothers. We prove in appendix B that given *β*_eff_(*a*), the new-mother birth rate function can be calculated from

2.19 $$\beta_{0}(a)=\frac{\beta_{\mathrm{eff}}(a)}{1-\int_{0}^{\delta }\beta_{\mathrm{eff}}(a-\tau )\;d\tau },$$

which then allows us to reconstruct *β*(*a*, *τ*) from equation (2.6). Using *β*_eff_, the boundary condition for equation (2.16), the counterpart to equation (2.15*c*), can be written as

2.20 $$h_{\mathrm{tot}}(0)=\eta \int_{0}^{\infty }\beta_{\mathrm{eff}}(a)h_{\mathrm{tot}}(a)\;da.$$

Finally, after using the solution in equation (2.17) for *h*_tot_(*a*) in equation (2.20), we find an equation for *λ*:

2.21 $$z(\lambda )\equiv \eta \int_{0}^{\infty }\beta_{\mathrm{eff}}(a)\exp\left[-a\lambda -\int_{0}^{a}\mu_{f}(a^{\prime })da^{\prime }\right]da=1.$$

The function *z*(*λ*) is monotonically decreasing with *λ* and obeys the limits lim _*λ*→+∞_ *z*(*λ*) = 0 and lim _*λ*→−∞_ *z*(*λ*) = +∞. Thus, equation (2.21) has a unique solution that can easily be found numerically. From equation (2.21), the solution for *λ*—the net population growth rate—clearly increases with *β*_eff_(*a*) and decreases with *μ*_f_(*a*).

With *β*_0_(*a*) and *λ* determined by equations (2.19) and (2.21), respectively, we can explicitly find *h*(*a*, *τ*). First, we use the normalization condition $\int_{0}^{\infty }h_{\mathrm{tot}}(a)da=1$ on equation (2.16) to explicitly find *h*_tot_(0) in terms of *λ* and *μ*_f_(*a*). Since *h*(0, *τ*) = 0, we have *h*_0_(0) = *h*_tot_(0), which allows us to explicitly express the solution to equation (2.15*a*):

2.22 $$h_{0}(a)=h_{0}(0)\exp\left[-a\lambda -\int_{0}^{a}(\mu_{f}(a^{\prime })+\beta_{0}(a^{\prime }))\;da^{\prime }\right].$$

Next, we use equation (2.15*d*) and equation (2.18) to eliminate *β*(*a*, *τ*) and find *h*(*a*, 0) = *h*_tot_(*a*)*β*_eff_(*a*), which is known. Thus, we can explicitly calculate *h*(*a*, *τ*) by solving equation (2.15*b*) using the method of characteristics:

2.23 $$h(a,\tau )=h(a-\tau ,0)\exp\left[-\tau \lambda -\int_{a-\tau }^{a}(\mu_{f}(a^{\prime })+\beta (a^{\prime },a^{\prime }-a+\tau ))\;da^{\prime }\right].$$

To summarize, starting from *β*_eff_(*a*), *μ*_f_(*a*), *η* (measured, say), and *δ*, we can compute the growth rate *λ* numerically, then analytically reconstruct *β*_0_(*a*), *β*(*a*, *τ*), *h*_0_(*a*) and *h*(*a*, *τ*).

We now use the Chinese national census data recorded in 1982 [20] to infer the overall birth rate *β*_eff_(*a*) and the female death rate *μ*_f_(*a*) functions in China in 1981. Although gestation imposes a hard refractory period of *δ* ≈ 9 months, some time is needed to recover from childbirth and the birth rate should more gradually recover. Specifically, in 1981 China, extended breastfeeding was common, which prevents the next pregnancy [27]. Thus, we will assume the birth rate returns to normal approximately only after about 2 years. Therefore, when there is no policy that controls the interval between births, we set δ=2 years such that *β*(*a*, *τ*) = 0 for *τ* < 2 years and *β*(*a*, *τ*) = *β*_0_(*a*) for τ>2 years. For other societies, this natural refractory period might be shorter. Using *δ* = 2 and equation (2.19), we calculate *β*_0_(*a*) from *β*_eff_(*a*) derived from data. These rates are illustrated in figure 1. Note that *β*_0_(*a*) for 1981 has already been mildly affected by the incipient birth-control policies in China.

**Figure 2.**
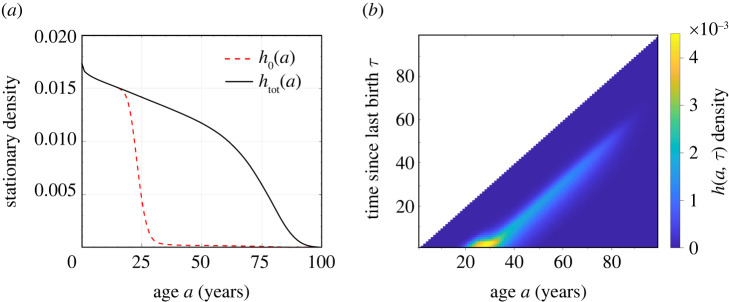
The asymptotic population distribution associated with the birth and death rates shown in figure 1. (*a*) Steady-state age distributions of females without children, *h*_0_(*a*), and all females, *h*_tot_(*a*), respectively. A monotonically decreasing *h*_tot_(*a*) indicates that there are larger numbers of younger females, consistent with an increasing population and *λ* > 0. (*b*) The full double density *h*(*a*, *τ*) of females of age *a* and whose youngest child is τ years  old.

**Figure 3.**
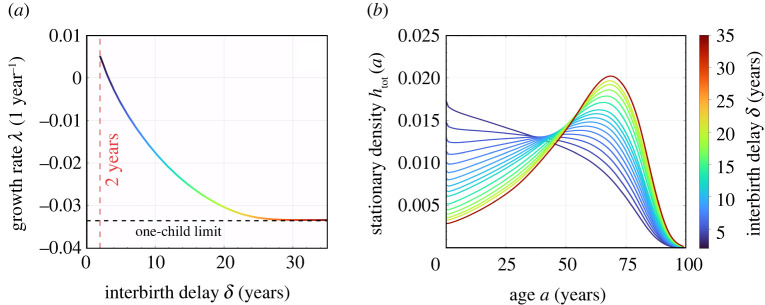
Asymptotic total-population growth rate and the steady-state, total-population age distribution. (*a*) Effect of an imposed interbirth delay *δ* on the asymptotic growth rate *λ*. Most of the decrease in *λ* occurs at small values of *δ* where the most negative slopes arise. When *δ* is large enough, a woman ages out before giving birth again, and imposing interbirth delay is equivalent to the strict one-child policy. (*b*) The steady-state age distribution *h*_tot_(*a*). As *δ* increases, the peak of population density moves from 0 to approximately 65.

Using the birth rate *β*_eff_(*a*) and death rate *μ*_f_(*a*) shown in figure 1, we solve equations (2.15) to find *h*_0_(*a*) and *h*(*a*, *τ*), and plot them with *h*_tot_(*a*) given by equation (2.17) in figure 2*a*,*b*. To explore the effects of an imposed refractory period, we first set δ=2 years, apply the newborn sex ratio of China in 1981, *η* = 0.48, and solve equation (2.21). We find *λ* ≃ 0.005 > 0, indicating an exponentially growing total population. This stationary growth rate is much smaller than the actual growth rate of China in 1981, which is 0.0146. One reason is that in 1981, the proportion of younger females is much higher than that in the stationary distribution *h*_tot_. The shape of *h*_tot_(*a*) is consistent with this growth as it is monotonically decreasing, indicating that every new generation has a larger population than the previous one. In figure 3, we see how increases in the refractory period *δ* decrease the asymptotic growth rate *λ* and affect the distribution *h*_tot_(*a*). A negative overall birth rate *λ* < 0 (i.e. an asymptotically decaying population) arises when δ≳3.22 years ≈39 months. As soon as *λ* < 0, the distribution *h*_tot_(*a*) becomes non-monotonic, and a peak in the female population distribution arises at a finite age *a* > 0.

If *δ* is set sufficiently large, a female cannot have a second child, and the outcome is equivalent to a strict one-child policy. We use the terminology ‘strict one-child policy’ for the scenario in which each female can have strictly no more than one child, while we use ‘one-child policy’ to refer to the actual policy realized in practice. From 1980 to 1990, the one-child policy contained many exceptions, allowing one to bear more than one child [28]. Our formulation is valid only in the asymptotic case with a fixed delay *δ* that remains unchanged for a long period of time. For practical modelling of policies in which delays *δ* are used as a time-dependent control variable, such as China’s 1980 one-child policy and its subsequent modification in 2015, it is necessary to analyse the full model that delineates the two female populations.

### 2.3. Temporal evolution

**Figure 4.**
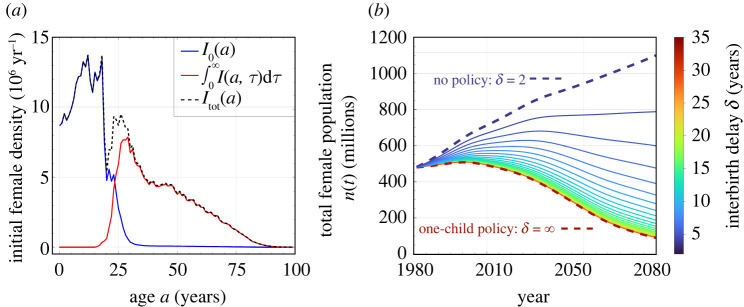
Evolution of the population for different delays. (*a*) Initial female subpopulation distributions in 1981 China (calculated from Population Census Office under the State Council [20]). (*b*) Evolution of the total female population *n*(*t*) for different imposed delays between births. These results are determined using the birth and death rates in figure 1 and the initial populations shown in (*a*). A relatively small delay between births (for example, δ∼4 years) has a significant impact on the evolution of *n*(*t*).

As was used to predict the effects of the one-child policy, we use China’s female age distribution in 1981 [20] as a starting point to explore how the total population evolves under different values of the imposed delay *δ*. Since the data only contain total female numbers *I*_tot_(*a*) and not *I*_0_(*a*) and *I*(*a*, *τ*) individually, we use *I*_0_(*a*) = *I*_tot_(*a*)*h*_0_(*a*)/*h*_tot_(*a*) and *I*(*a*, *τ*) = *I*_tot_(*a*)*h*(*a*, *τ*)/*h*_tot_(*a*) to reconstruct these initial age distributions. These initial distributions are plotted in figure 4*a*. With these initial conditions, we can solve equation (2.4*a*,*b*) with the method of characteristics to find the full age and time dependence of the female populations

2.24*a* $$f_{0}(t,a)=f_{0}(t-a,0)\exp\left[-\int_{0}^{a}(\mu_{f}(a^{\prime })+\beta_{0}(a^{\prime }))da^{\prime }\right]\;\text{if }t>a,$$

2.24*b* $$f_{0}(t,a)=I_{0}(a-t)\exp\left[-\int_{a-t}^{a}(\mu_{f}(a^{\prime })+\beta_{0}(a^{\prime }))da^{\prime }\right]\;\text{if}\;t\leq a,$$

2.25*a* $$f(t,a,\tau )=f(t-\tau ,a-\tau ,0)\exp\left[-\int_{a-\tau }^{a}(\mu_{f}(a^{\prime })+\beta (a^{\prime },a^{\prime }-(a-\tau )))da^{\prime }\right]\;\text{if}\;t>\tau$$

2.25*b* $$f(t,a,\tau )=I(a-t,\tau -t)\exp\left[-\int_{a-t}^{a}(\mu_{f}(a^{\prime })+\beta (a^{\prime },a^{\prime }-(a-\tau )))da^{\prime }\right]\;\text{if}\;t\leq \tau .$$

Females are fertile only between sexual maturity and menopause. Thus, we set *a*_min_ (approx. 12 years) and *a*_max_ (approx. 50 years), so that *β*_0_(*a*) = *β*(*a*, *τ*) = 0 for *a* < *a*_min_ or *a* > *a*_max_. Recall that an imposed delayed-birth policy is manifested by *β*(*a*, *τ*) = 0 for *τ* < *δ*. Equation (2.4*c*) becomes

2.26 $$f_{0}(t,0)=\eta \left(\int_{a_{\min}}^{a_{\max}}\;\beta_{0}(a)f_{0}(t,a)\;da+\int_{a_{\min}}^{a_{\max}}\;da\int_{\delta }^{a}\;d\tau \;\beta (a,\tau )f(t,a,\tau )\right).$$

For *t* ≤ *γ* ≡ min{*a*_min_, *δ*}, the *f*_0_(*t*, *a*) and *f*(*t*, *a*, *τ*) terms in the integrands in equation (2.26) can be solved by equation (2.24*b*) and equation (2.25*b*). Thus, we can express *f*_0_(*t*, 0) in terms of *I*_0_(*a*), *I*(*a*, *τ*), *β*_0_(*a*), *β*(*a*, *τ*) and *μ*_f_(*a*). Using equations (2.24), we can calculate *f*_0_(*t*, *a*) for any *a* and *t* ≤ *γ*. Under the imposed refractory period, equation (2.4*d*) becomes

2.27 $$f(t,a,0)=f_{0}(t,a)\beta_{0}(a)+\int_{\delta }^{a}\;\beta (a,\tau )f(t,a,\tau )\;d\tau .$$

If *t* ≤ *γ*, we can also use equation (2.25*b*) for *f*(*t*, *a*, *τ*) in the integrand of equation (2.27), and then use the solved *f*_0_(*t*, *a*) to express *f*(*t*, *a*, 0) in terms of *I*_0_(*a*), *I*(*a*, *τ*), *β*_0_(*a*), *β*(*a*, *τ*) and *μ*_f_(*a*). Using equations (2.25), we can calculate *f*(*t*, *a*, *τ*) for any *a*, *τ* and *t* ≤ *γ*. Finally, using *f*_0_(*γ*, *a*), *f*(*γ*, *a*, *τ*) as the initial conditions, we can solve *f*_0_(*t*, *a*), *f*(*t*, *a*, *τ*) for *t* ≤ 2*γ*. Repeating this procedure, we can use *I*_0_(*a*), *I*(*a*, *τ*), *β*_0_(*a*), *β*(*a*, *τ*) and *μ*_f_(*a*) to calculate *f*_0_(*t*, *a*) and *f*(*t*, *a*, *τ*) for any *t*, *a*, *τ*.

Using the fundamental rates *β*_0_(*a*), *μ*_f_(*a*) and *β*(*a*, *τ*) as those used in the previous subsection for the full model (see figure 1 and equation (2.6)), we use the above procedure to construct the total female population *n*(*t*) (see equation (2.3)). The evolution of *n*(*t*) over one century, under different interbirth delays, are plotted in figure 4*b*. At long times, the total population exhibits the asymptotic behaviour predicted by the eigenvalues shown in figure 3*a*. For δ≳3.22 years, the total population will decrease exponentially. Because the total population growth rate is most sensitive to small values of imposed delay *δ*, even a delay of δ∼4 years is sufficient to dramatically reduce population over the next 100 years, compared to the *δ* = 2 case of no refractory period.

The formal results and analyses above can be generalized to include time dependent parameters *μ*_f_(*t*, *a*), *β*_0_(*t*, *a*), *β*(*t*, *a*, *τ*), and even *δ*(*t*) to reflect social and policy changes. In this case, an imposed refractory period would be defined by the time-dependent birth rate function $\beta (t,a,\tau )=\beta_{0}(t,a)1(\tau >\delta (t))$ and the population densities will need to be evaluated numerically.

## 3. Results and discussion

Our basic structured population model can be modified and applied to different scenarios and policies to make predictions about a number of potentially relevant quantities. We focus on the population dynamics in China under different control scenarios, paying particular attention to age and sex distributions.

### 3.1. Predictions and comparison to data from China

**Figure 5.**
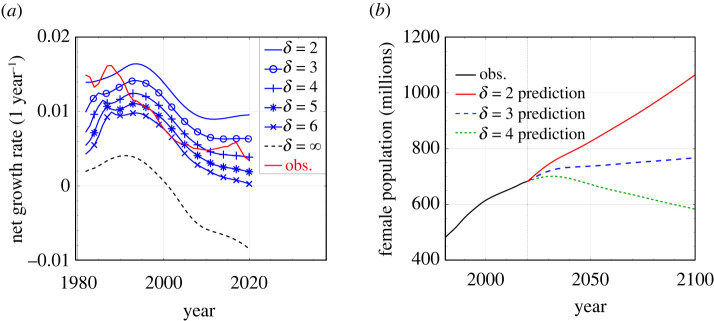
Predictions and comparison with real data. (*a*) Net growth rates in 1981–2020, observed (‘obs’) and predicted growth rates (starting from 1981 conditions) under different policies *δ*. (*b*) Predictions of the female population in 2021–2100 under different values of *δ*.

First, we use parameters inferred from 1981 Chinese data in our model to predict population growth for different values of *δ*. When we compare predicted net growth rates with those derived from 1981 to 2020 data, shown in figure 5*a*, we see that (i) during 1981–1990, the observed growth rate is close to that when *δ* = 2^1^; (ii) from 1991 to 2010, the observed growth rate was close to that predicted from a model with *δ* = 5, possibly due to harsher policies like coerced abortion [28]; (iii) after 2011, the observed growth rate rose to a level close to that of a model with *δ* = 3 ∼ 4, indicating a *de facto* relaxation of the one-child policy. Indeed, after 2011, policies that *encouraged* births were initiated. Starting in 2011, a couple was allowed to have up to two children if both parents never had siblings. Then, starting in 2014, a couple can have up to two children if at least one of the parents never had a sibling. Finally, starting in 2016, couples could bear up to two children regardless of their sibling status [30]. These policies might explain the flattening and subsequent increase in the net growth rate starting about 2011. However, the effects of these policies might be temporary. After the two-child policy in 2016, the net growth rate increased to the level consistent with a *δ* = 3 model but soon returned to the level closer a *δ* = 4 model. The actual growth rate is higher than in the *δ* = ∞, strict one-child policy model. This indicates that many couples had, legally or illegally, more than one child.

Starting in 2021, a new policy allows any couple to have up to three children without penalty. We make different predictions for the effect of this three-child policy. The optimistic prediction is that the policy can fully stimulate childbearing to the point that the net growth rate can reach the level predicted by a *δ* = 2 model, but this could be realized only if behaviour and cultural-economic changes have not affected an intrinsic propensity for childbearing. Another possibility is that the policy has no significant effect so that the net growth rate will only increase modestly and transiently before effectively reducing back to that consistent with a *δ* = 4 model. See figure 5*b* for different predictions of population over the 2021–2100 time frame.

### 3.2. Minimum childbearing age and population ageing

**Figure 6.**
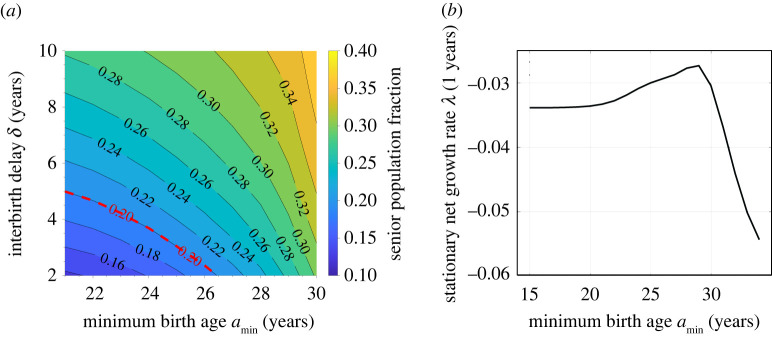
The effect of adjusting the minimum childbearing age. (*a*) Increasing the interbirth delay *δ* and increasing the minimum childbearing age *a*_min_ have similar side effects of increasing the percentage of senior population. The red dashed line indicates a dangerous senior population percentage, 20%. (*b*) Under the strict one-child policy (namely, *δ* = ∞), when we increase the minimum childbearing age *a*_min_, the stationary net growth rate *λ* will first increase then decrease.

Besides mandating a refractory period between births, another method of population control is to impose a minimum childbearing age. The minimum age *a*_min_ is thus set by policy rather than by physiology. For example, starting in 1985, in Yicheng county, Shanxi province, a couple could bear two children, but the first child was allowed only after the mother turned 24, and the second child was allowed only after the mother turned 30 [31].^2^ One side-effect of population control is a distribution shifted toward older ages. In China, the percentage of seniors (65+) increased from 5% in 1981 to 13% in 2020 [32]. Policies such as imposing interbirth delays *δ* and minimum childbearing ages *a*_min_ can both affect the long-term senior (65+) population. Figure 6*a* shows a contour plot of the percentage of seniors as a function of imposed *δ* and *a*_min_. In order to maintain the senior population under 20% (the red dashed line), the overall policy must not be too drastic. Nonetheless, with two strategies, a balanced combination can be used. For example, one can set *a*_min_ = 26, *δ* = 2, or *a*_min_ = 24, *δ* = 4, or *a*_min_ = 21, *δ* = 5 and still prevent the senior population from exceeding 20% far in the future.

Although increasing the minimum childbearing age *a*_min_ should reduce the rate of childbirth, we observe a counter-intuitive scenario in which the net growth rate is non-monotonic in *a*_min_. Under the strict one-child policy (i.e. *δ* = ∞), increasing *a*_min_ will first *increase* the stationary net growth rate *λ* before decreasing it, as shown in figure 6*b*. Under a perpetual strict one-child policy, the population in each successive generation is roughly halved and the total number of future newborns is roughly the current population. Although decreasing *a*_min_ can temporarily increase the total population, it accelerates the ‘halving process’ in the long run since the interval between successive generations is shorter. When *a*_min_ is not large, almost every woman can have one child anyway. Further increasing *a*_min_ will decrease *λ* as more women start to be pushed past their childbearing years without giving birth; thus, a maximum in the growth rate *λ* arises at *a*_min_ ≈ 29.

### 3.3. Female population fraction and interbirth delay

**Figure 7.**
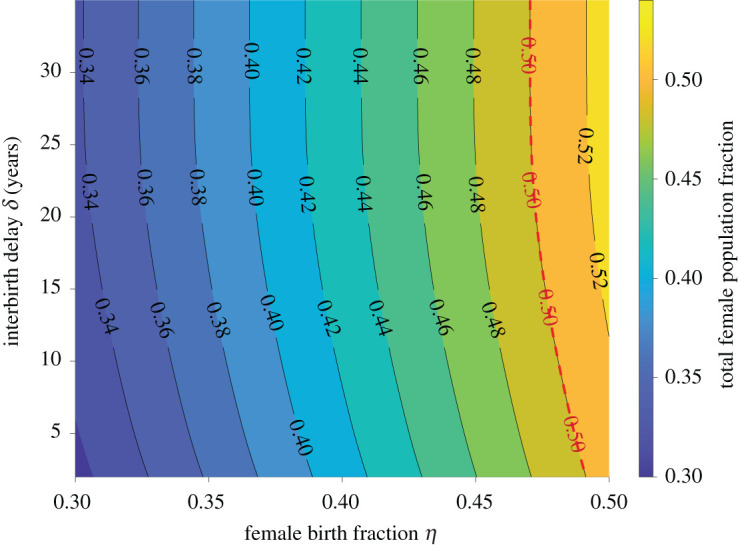
Dependence of the stationary female fraction on *η* and *δ*. Increasing the fraction of female births *η* and the interbirth delay *δ* can both increase the stationary female population fraction. The red dashed line indicates conditions for 50% females.

We used *η* = 0.48, the fraction of female births 1981 China, to generate the results presented in §2. Owing to subsequent sex-selective abortions biased towards males, the value of *η* dropped to 0.45 in 2005 before gradually increasing [24,33,34]. We can alter the value of *η* and calculate the stationary female fraction of total population, which is also affected by the interbirth delay *δ*. Figure 7 illustrates the stationary female percentage as a function of *η* and *δ*. When *δ* = 2, the stationary female percentage is approximately 1% higher than the female percentage *η* at birth. When we fix *η* and increase *δ*, the stationary female fraction also increases. When *δ* = ∞, the stationary female population is approximately 3% higher than *η*. For larger *δ*, the stationary age distribution is shifted to larger ages. Since the female death rate *μ*_f_(*a*) is lower than the male death rate *μ*_m_(*a*) at larger ages *a*, the female percentage increases with age (in 1981 China, newborns were 48% female while 65+ seniors were 56% female).

### 3.4. Behavioural response to policies

We have discussed the policy of applying a refractory period between births and predicted its effects. We assume that the birth rate returns to normal after the refractory period, meaning that people obey this policy and do not respond with compensatory behaviours. In reality, people who want to have more children might mitigate the effects of control policies by, for example, giving birth again immediately after the end of the refractory period following the previous birth. In addition to this ‘catch up’ strategy to recover from the ‘missed opportunity,’ people might also prefer to have the first child earlier, so that the refractory period finishes at a younger age.

We propose a model that considers possible behavioural responses and compare it with a no-response model. (1) For females of age *a* who have just finished their refractory period, the birth rate for the following year (only) will be set to *β*_0_(*a*)*c*_1_ instead of simply *β*_0_(*a*). We can model the compensatory increase of birth rate after the refractory period by *c*_1_ = 1 + 0.1 × min{*δ* − 2, 10} > 1. When the refractory period *δ* is longer, people are more likely to more quickly make up for the lost opportunity. (2) For females of age *a* who have not had children, if *a* + *δ* ≤ 40, the birth rate, instead of simply being *β*_0_(*a*), will be set to *β*_0_(*a*)*c*_2_, where *c*_2_ = 1 + 0.05 × min{*δ* − 2, 10}. This means that females prefer to have the first child earlier, if they know that they are young enough to have another child after the refractory period (the age will be *a* + *δ* at that future time).

**Figure 8.**
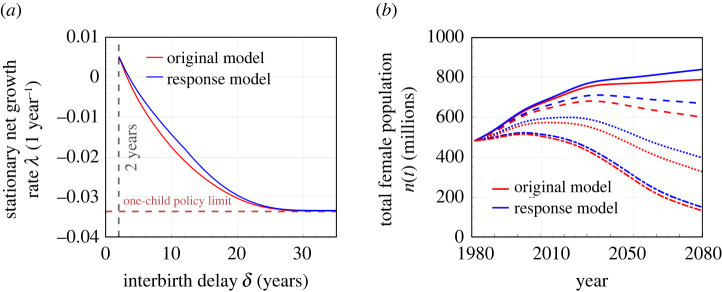
Comparison of the predictions of models with and without behavioural response. (*a*) Effect of an imposed interbirth delay *δ* on the asymptotic growth rate *λ*. The red curve depicts the no-response model, the same as the curve in figure 3*a*. The blue curve is the growth rate associated with the behavioural response model (where *β*_0_(*a*)*c*_1_ and *β*_0_(*a*)*c*_2_ are used as birth rates). For a moderate refractory period (*δ* = 5 ∼ 10), the behavioural response is equivalent to making *δ* 1 year shorter. (*b*) Evolution of the total female population *n*(*t*) for different imposed delays between births. The red and blue curves represent populations associated with the no-response (same as in figure 4*b*) and behavioural response models, respectively. The solid, dashed, dotted, dash-dotted curves correspond to δ=3,4,7,15 years, respectively. Behavioural responses have stronger effects on the total population when *δ* is not too large.

Figure 8 compares predictions from the standard no-response model (red) to those from a behavioural response model (blue). As expected, behavioural responses blunt the policy-induced decreases in the stationary growth rate (figure 8*a*) and the total population (figure 8*b*), resulting in higher-than-expected growth and populations. For an imposed *δ*, a compensatory behavioural response model leads to a higher stationary growth rate. In other words, if the behavioural responses of this example are included, the imposed delay *δ* would have to be about 1–2 years longer than in the absence of behavioural response in order to achieve the same overall growth rate (for intermediate delays δ≈5−15 years). However, if the refractory period is set very long (δ≳20 years), our proposed behavioural responses are futile since females are irreversibly moved past their fertility window.

### 3.5. Comparison between China and Japan

We have examined the effect of applying a refractory period policy in China, which has implemented various birth-control policies over past four decades. To better study this interbirth delay, we apply our model to Japan, which does not have enforced polices on population control. We use Japan’s 2000 population data as a starting point [35]. For Japan, we use its 2000 birth rate, which was much lower than that of 1981 China.

**Figure 9.**
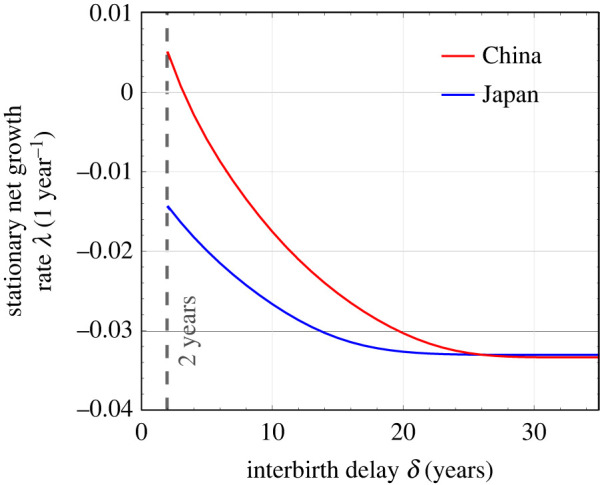
Effect of an imposed interbirth delay *δ* on the asymptotic growth rate *λ*, using birth rate data from China and Japan. The red curve is the asymptotic growth rate calculated from 1981 China data, the same as that shown in figure 3*a*. The blue curve is the asymptotic growth rate calculated from 2000 Japan data. Since Japan has a much lower birth rate, even without a refractory period policy (*δ* = 2), the stationary growth rate is negative. When *δ* is large enough, each female can have at most one child, and the asymptotic rates of population decay for China and Japan are nearly the same. However, under this large-*δ* sub-replacement limit, the long-term growth rate for Japan can actually be slightly larger (less negative) than that of China.

Figure 9 compares the stationary growth rates between China and Japan, imposing different refractory periods *δ*. Since Japan has a much lower growth rate *β*_0_, with the same *δ*, the stationary growth rate of Japan is lower than that of China. When *δ* is sufficiently large, since each female can have at most one child, the difference between China and Japan diminishes. In fact, the limiting high-*δ* stationary growth rate of Japan is slightly *higher* than that of China. Since the childbearing age is older in Japan, the gap between two successive generations is longer. As observed in figure 6*b*, under large-*δ*, sub-replacement conditions, a moderately longer gap between generations can increase the stationary (very long-term) growth rate.

## 4. Summary and conclusion

We have formulated a ‘continuum’ of birth-control policies for population management in which the strict one-child policy is a limiting case. Our approach is based on explicitly incorporating a refractory period between births. In general, our age- and gestation-period structured model can also apply to organisms in which the gestation time is appreciable compared to an organism’s window of fertility. For example, animals such as the Greater cane rat (*Thryonomys swinderianus*), the Pacarana (*Dinomys branickii*) and the Steenbok (*Raphicerus campestris*) have gestation periods approximately 15% of their fertility window [36]. Although a single gestation period is about 3% of the childbearing period in humans [37], socially imposed refractory periods can be much longer (and infinite in a strict one-child policy). Thus, our model provides a natural way to test how imposed tunable interbirth refractory periods *δ* affect the predicted total female population and its steady-state age distribution. For long delays *δ*, the model approaches the strict one-child policy as a larger fraction of women are pushed past menopause.

In our mathematical analysis, we found a number of analytic or closed-form solutions to relevant demographic quantities such as the steady-state age distribution. We then considered an alternative scenario in which ‘lax’ birth control policies that was being implemented in 1981 are kept, along with an additional policy of an imposed refractory period between births. Using 1981 as the starting point, we predicted population levels and compared them to the actual, realized populations. By applying a refractory period *δ* between births and using 1981 China birth rates, we provided a retrospective analysis and arrived at a number of quantitative conclusions. Our analyses assumed that the birth rate *β*_0_ and death rate *μ* did not change in the intervening years and that the population adhered to the birth-control policies without further behavioural responses. We concluded that: (i) when δ≳3.2 years, the total population will not grow in the long run (figure 3*a*); (ii) when δ≥4 years, the total population in China would have always been maintained under 1.45 billion (figure 4*b*); (iii) when δ≥6 years, the net growth rates during 1990–2010 (when a harsher one-child policy was applied) would be as low as what was realized (figure 5*a*); (iv) without increasing the minimum childbearing age, when δ≤5 years, the stationary senior population would be maintained under 20% (figure 6*a*).

Such predictions assume the adjusted birth rate *β*_0_(*a*) does not change over time. This assumption is definitely unrealistic, since many important socio-economic factors can affect birth rate distributions. The decrease of birth rate in China (illustrated in figure 4*a*) is not due solely to birth-control policies. After 1980, female education increased, which had the statistically significant effect of decreasing the birth rate [38]. Additional evidence consistent with an extra-policy influence on birth rates in China is the increase in the average age of first childbirth (which one expects to be less affected by policies) from 24.3 years to 26.9 years from 2006 to 2016 [39]. Moreover, we expect behavioural responses to policies that could mitigate their effectiveness. Since it is difficult to separate and quantify the effects of socio-economic factors and behavioural responses on birth rates, we did not explicitly incorporate these factors in our model. Nonetheless, we discussed how policies can be implemented through different modifications of age- and refractory period-dependent birth rate functions. For example, we considered a population-control policy whereby a minimum birth age *a*_min_ is imposed. Here, we found the counterintuitive result that under a strict one-child policy, increasing *a*_min_ first *increases* the stationary net growth rate, before decreasing it as *a*_min_ is further increased.

Age-structured models can also be generalized to include additional subpopulations, such as those arising in cell division [40] and disease propagation [41] models. For example, in the birth control context, different generations and family structure can be enumerated in order to predict the effects of policies such as those implemented in 2011 and 2014 that consider the sibling status of would-be parents, allowing those without siblings more latitude in childbirth. Additional concepts from sociology and response to socioeconomic and political influences can also potentially be integrated for a more complete framework of population dynamics and demography. The ideas and mathematical tools in this paper can be adapted to other fields. For example, an economist or a sociologist might study the cultural norms regarding child spacing and use our models to connect child spacing to growth rates.

## Data Availability

Data and relevant code for this research work are stored in GitHub: https://github.com/YueWangMathbio/ChildPolicy and have been archived within the Zenodo repository: https://doi.org/10.5281/zenodo.6394805 [42].

## Footnotes

1 This means that there was effectively no interbirth period policy and that the adjusted birth rate *β*_0_(*a*) was not changed much during the lax birth-control policies during this period [29].

2 In China, the legal marriage age for females is 20 implying an existing soft constraint of *a*_min_ ≈ 21.

## Appendix A. The equation of *f*_tot_(*t*, *a*)

We show by direct substitution that the total female population density *f*_tot_(*t*, *a*) satisfies the standard age-structured McKendrick equation. Substitution of equation (2.2) into equation (2.5) and expanding,

A 1 $$\begin{array}{rl} \frac{\partial }{\partial t}f_{\mathrm{tot}}(t,a)+\frac{\partial }{\partial a}f_{\mathrm{tot}}(t,a) & =\frac{\partial }{\partial t}f_{0}(t,a)+\frac{\partial }{\partial a}f_{0}(t,a)\\ & \;+\int_{0}^{a}\frac{\partial }{\partial t}f(t,a,\tau )\;d\tau +\int_{0}^{a}\frac{\partial }{\partial a}f(t,a,\tau )\;d\tau \\ & \;+f(t,a,a)+\int_{0}^{a}\frac{\partial }{\partial \tau }f(t,a,\tau )d\tau -\int_{0}^{a}\frac{\partial }{\partial \tau }f(t,a,\tau )\;d\tau \\ & =-(\mu_{f}(a)+\beta_{0}(a))f_{0}(t,a)-\int_{0}^{a}(\mu_{f}(a)+\beta (a,\tau ))f(t,a,\tau )\;d\tau \\ & \;+f(t,a,a)-f(t,a,a)+f(t,a,0)\\ & =-\mu_{f}(a)f_{\mathrm{tot}}(t,a)-\beta_{0}(a)f_{0}(t,a)-\int_{0}^{a}\beta (a,\tau )f(t,a,\tau )\;d\tau \\ & \;+\beta_{0}(a)f_{0}(t,a)+\int_{0}^{a}\beta (a,\tau )f(t,a,\tau )\;d\tau \\ & =-\mu_{f}(a)f_{\mathrm{tot}}(t,a). \end{array}$$

## Appendix B. Relation between *β*_eff_(*a*) and *β*_0_(*a*)

In this appendix, we prove equation (2.19), the link between *β*_eff_(*a*) and *β*_0_(*a*). In the following, assume *τ* ≤ *δ*. In equation (2.23), *β*(*a*′, *a*′ − *a* + *τ*) = 0 for any *a* − *τ* < *a*′ < *a*. Thus, we have

B 1 $$\frac{h(a,\tau )}{h(a-\tau ,0)}=\exp\left[-\tau \lambda -\int_{a-\tau }^{a}\mu_{f}(a^{\prime })\;da^{\prime }\right],$$

while from equation (2.17), we have

B 2 $$\frac{h_{\mathrm{tot}}(a)}{h_{\mathrm{tot}}(a-\tau )}=\exp\left[-\tau \lambda -\int_{a-\tau }^{a}\mu_{f}(a^{\prime })\;da^{\prime }\right].$$

Thus,

B 3 $$\frac{h_{\mathrm{tot}}(a)}{h_{\mathrm{tot}}(a-\tau )}=\frac{h(a,\tau )}{h(a-\tau ,0)}.$$

From equations (2.15*d*) and (2.18), we have

B 4 $$h(a-\tau ,0)=\beta_{\mathrm{eff}}(a-\tau )h_{\mathrm{tot}}(a-\tau ).$$

Upon combining equations (B 3) and (B 4), we arrive at

B 5 $$h(a,\tau )=\beta_{\mathrm{eff}}(a-\tau )h_{\mathrm{tot}}(a).$$

Since equation (B 5) is valid for any *τ* ≤ *δ*, we have

B 6 $$\int_{0}^{\delta }\beta_{\mathrm{eff}}(a-\tau )d\tau =\frac{\int_{0}^{\delta }h(a,\tau )d\tau }{h_{\mathrm{tot}}(a)}.$$

Equation (2.18) can be transformed into

B 7 $$\beta_{\mathrm{eff}}(a)=\beta_{0}(a)\frac{h_{0}(a)+\int_{\delta }^{a}\;h(a,\tau )d\tau }{h_{\mathrm{tot}}(a)}=\beta_{0}(a)\left[1-\frac{\int_{0}^{\delta }h(a,\tau )\;d\tau }{h_{\mathrm{tot}}(a)}\right].$$

Combining equations (B 6) and (B 7), we obtain $\beta_{\mathrm{eff}}(a)=\beta_{0}(a)[1-\int_{0}^{\delta }\beta_{\mathrm{eff}}(a-\tau )\;d\tau ]$ and thus

B 8 $$\beta_{0}(a)=\frac{\beta_{\mathrm{eff}}(a)}{1-\int_{0}^{\delta }\beta_{\mathrm{eff}}(a-\tau )\;d\tau }.$$

## References

[1] Kermack WO, McKendrick AG. 1927 Contributions to the mathematical theory of epidemics. I. Proc. R. Soc. Lond. A **115**, 700-721. (10.1016/S0092-8240(05)80040-0)

[2] McKendrick AG. 1926 Applications of mathematics to medical problems. Proc. Edinburgh Math. Soc. **44**, 98-130. (10.1017/S0013091500034428)

[3] von Foerster H. 1959 Some remarks on changing populations in the kinetics of cell proliferation. Berlin, Germany: Springer.

[4] Keyfitz BL, Keyfitz N. 1997 The McKendrick partial differential equation and its uses in epidemiology and population study. Math. Comput. Modell. **26**, 1-9. (10.1016/S0895-7177(97)00165-9)

[5] Perthame B. 2007 Transport equations in biology. Basel, Switzerland: Birkhäuser Basel.

[6] Jiang D-Q, Wang Y, Zhou D. 2017 Phenotypic equilibrium as probabilistic convergence in multi-phenotype cell population dynamics. PLoS ONE **12**, e0170916. (10.1371/journal.pone.0170916) 28182672 PMC5300154

[7] Chou T, Greenman CD. 2016 A hierarchical kinetic theory of birth, death andfission in age-structured interacting populations. J. Stat. Phys. **164**, 49-76. (10.1007/s10955-016-1524-x) 27335505 PMC4894939

[8] Greenman CD. 2017 A path integral approach to age dependent branching processes. J. Stat. Mech: Theory Exp. **2017**, 033101. (10.1088/1742-5468/aa4f16)

[9] Greenman CD, Chou T. 2016 A kinetic theory for age-structured stochastic birth-death processes. Phys. Rev. E **93**, 012112. (10.1103/PhysRevE.93.012112) 26871029

[10] Xia M, Chou T. 2021 Kinetic theory for structured populations:application to stochastic sizer-timer models of cell proliferation. J. Phys. A: Math. Theor. **54**, 385601. (10.1088/1751-8121/abf532)

[11] Bongaarts J, Greenhalgh S. 1985 An alternative to the one-child policy in China. Popul. Dev. Rev. **11**, 585-617. (10.2307/1973456)

[12] Feeney G, Yu J. 1987 Period parity progression measures of fertility in China. Popul. Stud. **41**, 77-102. (10.1080/0032472031000142546) 11612059

[13] Tuljapurkar S. 1983 Transient dynamics of yeast populations. Math. Biosci. **64**, 157-167. (10.1016/0025-5564(83)90001-9)

[14] Falkenburg DR. 1973 Optimal control in age dependent populations. In *Joint Automatic Control Conf.*, Columbus, OH, 20–22 June 1973, pp. 112–117. New York, NY: IEEE.

[15] Hritonenko N, Yatsenko Y. 2010 Age-structured PDEs in economics, ecology, and demography: optimal control and sustainability. Math. Popul. Stud. **17**, 191-214. (10.1080/08898480.2010.514851)

[16] Langhaar HL. 1972 General population theory in the age-time continuum. J. Franklin Inst. **293**, 199-214. (10.1016/0016-0032(72)90085-3)

[17] Pollard JH. 1973 Mathematical models for the growth of human population. Cambridge, UK: Cambridge University Press.

[18] Song J. 1980 Bilinear optimal control with constraints in population systems (in Chinese). Zidonghua Xuebao **6**, 241-249. 12265334

[19] Song J. 1982 Some developments in mathematical demography and their application to the People’s Republic of China. Theor. Popul. Biol. **22**, 382-391. (10.1016/0040-5809(82)90051-X) 7163980

[20] Population Census Office under the State Council, editor. 1985 *1982 Population Census of China (in Chinese)*. Beijing, China: China Statistics Press.

[21] Song J, Kong D, Yu J. 1988 Population system control. Math. Comput. Modell. **11**, 11-16. (10.1016/0895-7177(88)90443-8)

[22] Bacaër N. 2011 A short history of mathematical population dynamics. Berlin, Germany: Springer Science & Business Media.

[23] Greenhalgh S. 2004 Science, modernity, and the making of China’s one-child policy. Popul. Dev. Rev. **29**, 163-196. (10.1111/j.1728-4457.2003.00163.x)

[24] Li H, Meng L. 2014 High sex ratio in china: Causes and consequences. In *The Oxford companion to the economics of China* (eds S Fan, R Kanbur, S-J Wei, X Zhang), ch. 81. Oxford Scholarship Online.

[25] Gurtin M, MacCamy RC. 1974 Non-linear age-dependent population dynamics. Arch. Ration. Mech. Anal. **54**, 281-300. (10.1007/BF00250793)

[26] Arino O. 1995 A survey of structured cell population dynamics. Acta Biotheor. **43**, 3-25. (10.1007/BF00709430) 7709687

[27] Xu F, Qiu L, Binns CW, Liu X. 2009 Breastfeeding in China: a review. Int. Breastfeed. J. **4**, 1-15. (10.1186/1746-4358-4-1) 19531253 PMC2706212

[28] Nie J-B. 1999 The problem of coerced abortion in China and related ethical issues. Camb. Q Healthc. Ethics **8**, 463-475. (10.1017/S0963180199004077) 10513303

[29] Li W, Wong W. 2020 Advocacy coalitions, policy stability, and policy change in China: the case of birth control policy, 1980–2015. Policy Stud. J. **48**, 645-671. (10.1111/psj.12329)

[30] Kane D, Li K. 2021 Fertility cultures and childbearing desire after the two-child policy: evidence from southwest China. J. Family Stud. 1-19. (10.1080/13229400.2021.1951813)

[31] Qin Y, Wang F. 2017 Too early or too late: what have we learned from the 30-year two-child policy experiment in Yicheng, China? Demogr. Res. **37**, 929-956. (10.4054/DemRes.2017.37.30)

[32] Man W, Wang S, Yang H. 2021 Exploring the spatial-temporal distribution and evolution of population aging and social-economic indicators in China. BMC Public Health **21**, 1-13. (10.1186/s12889-020-10013-y) 34020620 PMC8140474

[33] Goodkind D. 2011 Child underreporting, fertility, and sex ratio imbalance in China. Demography **48**, 291-316. (10.1007/s13524-010-0007-y) 21336689

[34] Zeng Y, Tu P, Gu B, Xu Y, Li B, Li Y. 1992 Sex ratio of China’s population deserves attention. China Popul. Today **9**, 3-5. 12318232

[35] Statistics Bureau of Japan (editor). 2001 2000 Population Census of Japan (in Japanese). Tokyo, Japan: Japan Statistical Society.

[36] Woods CA, Kilpatrick CW. 2005 Infraorder hystricognathi. In *Mammal species of the world: A taxonomic and geographic reference* (eds DE Wilson, DM Reeder), 3rd edn., p. 1538. Baltimore, MD: Johns Hopkins University Press.

[37] Tacutu R et al. 2018 Human ageing genomic resources: new and updated databases. Nucleic Acids Res. **46**, D1083-D1090. (10.1093/nar/gkx1042) 29121237 PMC5753192

[38] Lan M, Kuang Y. 2016 The impact of women’s education, workforce experience, and the one child policy on fertility in China: a census study in Guangdong, China. Springerplus **5**, 1-12. (10.1186/s40064-015-1659-2) 27777846 PMC5050180

[39] He D, Zhang X, Zhuang Y, Wang Z, Jiang Y. 2019 China fertility report, 2006–2016. China Popul. Dev. Stud. **2**, 430-439. (10.1007/s42379-019-00022-9)

[40] Xia M, Greenman CD, Chou T. 2020 PDE models of adder mechanisms in cellular proliferation. SIAM J. Appl. Math. **80**, 1307-1335. (10.1137/19M1246754) 35221385 PMC8871769

[41] Kang H, Ruan S. 2021 Mathematical analysis on an age-structured SIS epidemic model with nonlocal diffusion. J. Math. Biol. **83**, 1-30. (10.1007/s00285-021-01634-x) 34173884 PMC8234772

[42] Wang Y, Dessalles R, Chou T. 2022 Data from: Modelling the impact of birth control policies on China's population and age: effects of delayed births and minimum birth age constraints. Dryad Digital Repository. (10.5281/zenodo.6394805) PMC9174737 35706677

